# Effect of Parent‐Based Sexual Health Education on Parent–Adolescent Communication and Adolescent Sexual Behavior: A Systematic Review and Meta‐Analysis

**DOI:** 10.1111/psrh.70029

**Published:** 2025-08-05

**Authors:** Birhanu Gutu, Abela Mahimbo, Nikki Percival, Daniel Demant

**Affiliations:** ^1^ School of Public Health, Faculty of Health University of Technology Sydney Sydney Australia; ^2^ Department of Public Health Dambi Dollo University Dambi Dollo Ethiopia; ^3^ School of Public Health and Social Work, Faculty of Health Queensland University of Technology Brisbane Australia

**Keywords:** adolescent, health promotion, parent–adolescent communication, parent‐based sexual health education intervention, reproductive health, sexual health

## Abstract

**Background:**

Parent‐based sexual education interventions have a positive impact on adolescent sexual and reproductive health. However, despite these positive outcomes, there is a lack of comprehensive research to understand the effectiveness of these programs across different communities and demographics.

**Methods:**

We conducted a systematic search of databases from January 2013 to April 2023 and identified 51 published studies conducted globally on the effects of parent‐based sexual education. We conducted meta‐analyses to understand the pooled effect of interventions on parent–adolescent communication outcomes and adolescent sexual and reproductive behaviors.

**Results:**

This review includes 51 studies. Most of the included studies (*n* = 36, 68.6%) were conducted in high‐income countries; 17 (33.3%) involved only mothers, and 37 (72.5%) stated they were based on theoretical frameworks. The systematic review showed that interventions on sexual communication had mixed effects on parent and adolescent‐related outcomes. The meta‐analysis, however, showed positive effects on parent‐reported sexual health communication frequency, adolescent‐reported sexual health communication frequency, parental attitude, and parental self‐efficacy, Cohen's *d* = 0.32, 0.26, 0.38, and 0.41, respectively.

**Conclusion:**

Parent‐based sexual health education interventions positively impact various aspects of parent–adolescent sexual health communication and behavior, suggesting the effectiveness of the intervention in different social, cultural, and economic contexts. The lack of research in low‐ and middle‐income nations and limited paternal participation highlights the need for further research.

## 1 Introduction

Adolescence is defined by the World Health Organization (WHO) as the age between 10 and 19 years [1]. Adolescence is a critical period during which most adolescents initiate sexual activities [2, 3, 4] and start to explore their identities [5]. Appropriate support and guidance from parents are important at this stage to help adolescents navigate the physical, emotional, and cognitive changes they experience more effectively. Such support may enhance safe sexual behaviors, potentially leading to increased protection against problems associated with sexual risk behavior [6]. Providing parents with the tools they need to step in appropriately at this point of their child's sexual development could, therefore, be beneficial [7].

### 1.1 The Role of Parents in Sexual Health Education

Research has shown that parent‐based interventions for sexual health education significantly influence adolescents' sexual decision‐making [8, 9, 10, 11]. Parents, guardians, and caregivers are crucial information sources and are uniquely positioned to impart values, beliefs, expectations, and knowledge to young people [12]. Adolescents also rely on their parents for guidance and information regarding sexual health, making them influential figures in adolescents' sexual decision‐making process [13]. As such, parent–adolescent sexual health communication has been generally accepted as a pathway of reducing sexual risk behaviors among adolescents [14].

By providing accurate information about sexual health, parents can help their children make informed decisions and develop positive attitudes toward relationships and sexuality. This role of the parents as educators for adolescents in sexual health is supported by research [10] and strengthened by global policies such as the International Technical Guidance on Sexuality Education in Europe, developed by the United Nations Educational, Scientific and Cultural Organization (UNESCO) [15, 16].

### 1.2 Impact of Parent–Adolescent Communication

The WHO defines sexual health as “a state of physical, emotional, mental and social well‐being in relation to sexuality; it is not merely the absence of disease, dysfunction or infirmity” [17], whereas reproductive health is referred to as “a state of complete physical, mental and social well‐being and not merely the absence of disease or infirmity, in all matters relating to the reproductive system and its functions and processes” [18]. In this context, sexual and reproductive health (SRH) communication refers to interpersonal communication about SRH issues that takes place between parents or primary caregivers and adolescents [19]. Parent‐based sexual health education intervention in our study contextually refers to any intervention that aims to provide information and the required skills to parents and adolescents to improve parent–adolescent sexual health communication and empower adolescents to practice safe sex, prevent sexually transmitted infections (STIs) and avoid unplanned pregnancies [20].

Comprehensive sexuality education (CSE) is crucial for promoting SRH among adolescents [21]. Involving parents in this educational process can enhance its effectiveness [22], providing more personalized and impactful learning experiences for young people [10]. Evidence indicates that parent‐based interventions have been shown to enhance the frequency of parent–child communication about sexual health [23, 24]. Improved quality and self‐efficacy in SRH communication can influence behavioral change, as evidenced by the information communicated and participants' motivation to communicate [25]. Such a change in communication in turn fosters safer sex behaviors [26, 27], particularly for girls, and is more impactful when it occurs with mothers rather than fathers [28]. The relationship between mothers and daughters is particularly impactful for girls for several key reasons. Although mothers can serve as role models for girls, influencing their self‐esteem [29], societal expectations place mothers in the role of emotional support providers, creating a more open environment for girls to discuss emotions with their mothers [30]. Additionally, mothers may communicate in ways that resonate more with girls, fostering comfort and expression [31].

Furthermore, parent‐based sexual health education interventions have been associated with increased confidence and openness to sexual communication [9] and demonstrated positive changes in parental attitudes toward sexuality [32, 33]. As an example, a study conducted in Ghana showed a 30% increase in parents' positive attitudes toward adolescents' sexuality in the intervention group compared to the control group [20].

By positively affecting parents' perspectives on sexual health discussions, parent‐based sexual health education has the potential to improve adolescents' SRH outcomes [32, 34]. These include a reduction in the chance of becoming infected with STIs or experiencing an unplanned pregnancy [35].

However, some research reported mixed effects from parent‐based sexual health education interventions. Although some studies indicated improvement in parent‐reported communication frequency [8, 9, 11] and improved parent‐reported self‐efficacy [8, 9, 36] other studies indicated no statistically significant effect of parent‐reported sexual communication frequency [37] and self‐efficacy [38] and adolescent‐reported multiple sexual partners [11, 39]. This variation in the reported effectiveness of interventions might depend on their type, delivery method, duration, and other contextual factors, such as the social, cultural, and economic context, in which they are used and their quality. Additionally, these studies have not covered the impact of parental involvement programs on adolescent pregnancy and abortion rates.

### 1.3 Gaps in Current Research

Despite the existence of a reasonably well‐developed body of research on parental communication, variations were observed in their effect, and there is yet little information on the overall impact of such interventions, comparing the effects across various social, cultural, and economic contexts to guide future policy, practice, and research. A few previously published reviews [20, 40, 41, 42] provided information about the effectiveness of parent‐based sexual health education interventions. However, these were limited to the United States (US) and, while helpful, are situated within the US–American context of sexuality and parenting that may not be (fully) applicable to other contexts, particularly for countries with a comparably high prevalence of HIV and other STIs [43] or different perceived ethics and morals around sex and sexuality [44]. A systematic review of internationally conducted studies [45] is constrained by its scope as it only included qualitative studies.

This review, therefore, aims to comprehensively synthesize evidence using a systematic review and meta‐analysis on the aggregate effect of parent‐based sexual health education from globally accessible contemporary interventional research. The review compared the effectiveness of parent‐based sexual health education interventions across different social, cultural, and economic contexts to answer the overall research questions for this study:

What are the effects of parent‐based sexual health education interventions on parent–adolescent communication and adolescent SRH behaviour and outcomes?

The findings of this review may help inform policy, practice, and future research at both global and national levels.

## 2 Methods

### 2.1 Eligibility Criteria

The researchers conducted this systematic review following the Preferred Reporting Items for Systematic Reviews and Meta‐Analysis (PRISMA) Statement [46], with a detailed checklist provided in Supporting Information: Annex 1. We considered studies eligible for inclusion if they were published in peer‐reviewed journals, published in English, between January 2013 and April 2023. We included a variety of studies: randomized controlled trials (RCT), cluster randomized controlled trials (cRCT), and non‐randomized studies for intervention (NRSI). We also included all studies that involved participants who were either biological parents or primary caregivers and had at least one adolescent aged between 10 and 19 years, which made them eligible for our review. We incorporated studies regardless of their settings and countries as we aimed to compare the effect of the interventions across settings and countries' economic contexts.

We deemed parent‐based sexual health education intervention studies that measured at least one aspect of the parent‐reported outcomes: communication frequency, parental expected outcome, parental comfort, parental attitude, and parental self‐efficacy, as well as an adolescent reported outcome: consistent condom use, sexual initiation, multiple sexual partners, unintended pregnancy, and abortion as eligible for our review.

### 2.2 Definition of Key Concepts

Below are the definitions of key outcome variables in our study. We defined these variables based on existing literature, while also tailoring them to fit our specific research context. These definitions helped us to measure the variables accurately, determine the appropriate analysis methods, and present the results and discussion consistently.

#### 2.2.1 Parent–Adolescent Sexual Health Communication Frequency

The frequency with which parents and adolescents report discussing SRH topics with each other [37, 47].

#### 2.2.2 Parental Attitude

The beliefs, feelings, and behaviors that parents hold regarding communication about SRH with their adolescents [48, 49].

#### 2.2.3 Parental Self‐Efficacy

A perceived capability to perform a behavior [50]. Parental self‐efficacy, in our context, refers to a parent's belief in their ability to have SRH conversations with their adolescents.

#### 2.2.4 Parental Outcome Expectation

Parents' assessment of the results of having SRH conversations with their adolescents. This definition was based on Bandura's definition of outcome expectation. Bandura defines an outcome expectation as a judgment of the likely consequences that result from the performance of a behavior [51].

#### 2.2.5 Multiple Sexual Partners

Denotes having sexual intercourse with two or more sexual partners since sexual initiation [52].

#### 2.2.6 Consistent Condom Use

Represents consistent use as reporting condom use at every sexual encounter in a given period [53].

#### 2.2.7 Sexual Debut

Reflects having a first‐time sexual intercourse, which can be penile vaginal, oral, or anal [54, 55].

#### 2.2.8 Adolescent Pregnancy

Denotes pregnancy in a person aged 10–19 years [56].

#### 2.2.9 Abortion

Refers to the intentional discontinuation of a pregnancy [57].

#### 2.2.10 Organization‐Based Settings

Contextualized as physical workplaces, like business organizations, health care settings, or others.

### 2.3 Search Methods for the Identification of Studies

We searched the studies in different and relevant databases, including CINAHL/EBSCO, Scopus, MEDLINE/OVID, PsycINFO/EBSCO, and EMBASE/OVID. We selected these databases based on their relevance to the review topic and the high volume of health‐related journals indexed in these databases [58, 59]. These databases have also been used in previous systematic reviews and meta‐analyses on similar topics [20, 25, 26]. We provided the full search strategies for CINAHL/EBSCO, EMBASE/OVID, MEDLINE/OVID, PsycINFO/EBSCO, and Scopus in Supporting Information: Annexes 2–6, respectively.

### 2.4 Data Collection and Analysis

We utilized the Covidence software [60] to screen the articles and remove duplicates. We used parent‐based sexual health education intervention, adolescents 10–19 years old, and the year of publication from January 2013 to April 2023 as screening criteria in the title and abstract. Based on the criteria, B.G. screened the titles and abstracts of the articles to see if they were eligible for full‐text review. N.P., A.M., and D.D. independently reviewed a random sample of 100 articles and validated the screening. B.G. then conducted the full‐text review, and D.D., A.M., and N.P. double‐checked the full‐text review to ensure the validity. We collected detailed information on the characteristics of participants (both parents and adolescents), including mean age, sex, settings, country, number of interventions, and controls. We also investigated the methodology, such as study design, sample size, sampling methods, and recruitment methods. Additionally, we examined information about the interventions, including a description of interventions, setting, method of delivery, frequency, dose, and duration. We also recorded information about outcome measures, such as the timing and frequencies, effect estimate (point estimates and measures of variability, frequency counts for dichotomous variables), and effect size. B.G. extracted the data, and N.P., A.M., and D.D. checked its accuracy. We solved conflicts by discussion and consultation. When the included articles presented multiple post‐intervention measurements for any review outcomes, we selected the most recent measure for comparison [61].

### 2.5 Unit of Analysis

We conducted the analysis at the study level for all studies. For cRCT, we calculated effective sample sizes (ESS) [62] by adjusting the original sample size using a design effect based on an assumed intraclass correlation coefficient (ICC) of 0.02 [63].

### 2.6 Data Synthesis

We used both narrative and statistical synthesis and used an intention‐to‐treat approach in meta‐analysis. All studies were included in the qualitative synthesis, and studies with raw data were included in the meta‐analysis. The treatment effects were measured by Cohen's *d* using a random effect model for continuous outcomes. Pre‐calculated effect data and standard errors were used to calculate the log risk ratio (RR) for multiple sexual partners in adolescents. Standard errors were calculated from a 95% confidence interval, the upper limit minus the lower limit, and divided by 3.92. We classified the effect sizes as small (*d* = 0.2), medium (*d* = 0.5), and large (*d* ≥ 0.8) [64]. The Statistical Package for Social Sciences (SPSS) 28 [65] was used for meta‐analyses. We used a random‐effect model with an inverse‐variance method with a minimum of three eligible articles to conduct the meta‐analysis for continuous outcomes. A random‐effect model for non‐continuous outcomes was used.

### 2.7 Assessment of Heterogeneity

We assessed heterogeneity using a Forest plot and *I*‐square (*I* ^2^) test and interpreted the *I* ^2^ as 0%–40%: might not be important; 30%–60%: moderate heterogeneity; 50%–90%: substantial heterogeneity; and 75%–100%: considerable heterogeneity. The importance of the observed value of *I* ^2^ depends on (1) the magnitude and direction of effects, and (2) the strength of evidence for heterogeneity (e.g., *p* value from the Chi^2^ test, or a confidence interval for *I* ^2^: uncertainty in the value of *I* ^2^ is substantial when the number of studies is small) [66]. To assess the source of heterogeneity, we carried out subgroup analyses for study designs, intervention settings, the economic status of the countries, and the application of theory.

### 2.8 Assessment of Risk of Bias in Included Studies

We employed the revised Cochrane risk of bias tool for randomized trials (RoB2) [67], RoB2 for cluster‐randomized trials [68] and Risk of Bias in Non‐Randomized Studies of Interventions (ROBINS‐I) [69] to assess the risk of bias in RCTs, cRCTs, and NRSIs, respectively. We made judgments for domain‐level bias and overall risk of bias as “low risk,” “high risk,” or “some concern.” We assessed risk of bias based on information included in the publications of included studies without taking further information (e.g., from websites) into account, and the results should be interpreted within this context. Most of the outcomes were found to have a high risk of bias in the overall domain. Supporting Information: Annexes 7–9 contain the risk of bias assessment for each of the outcomes.

### 2.9 Assessment of Reporting Biases

We evaluated publication bias assessment using both Funnel's plot and Egger's regression‐based test. We used Funnel's plot to visualize the symmetry of the distribution, and a *p* value < 0.05 to indicate possible publication bias in Egger's test [70].

## 3 Results

### 3.1 The Result of the Search

**FIGURE 1:**
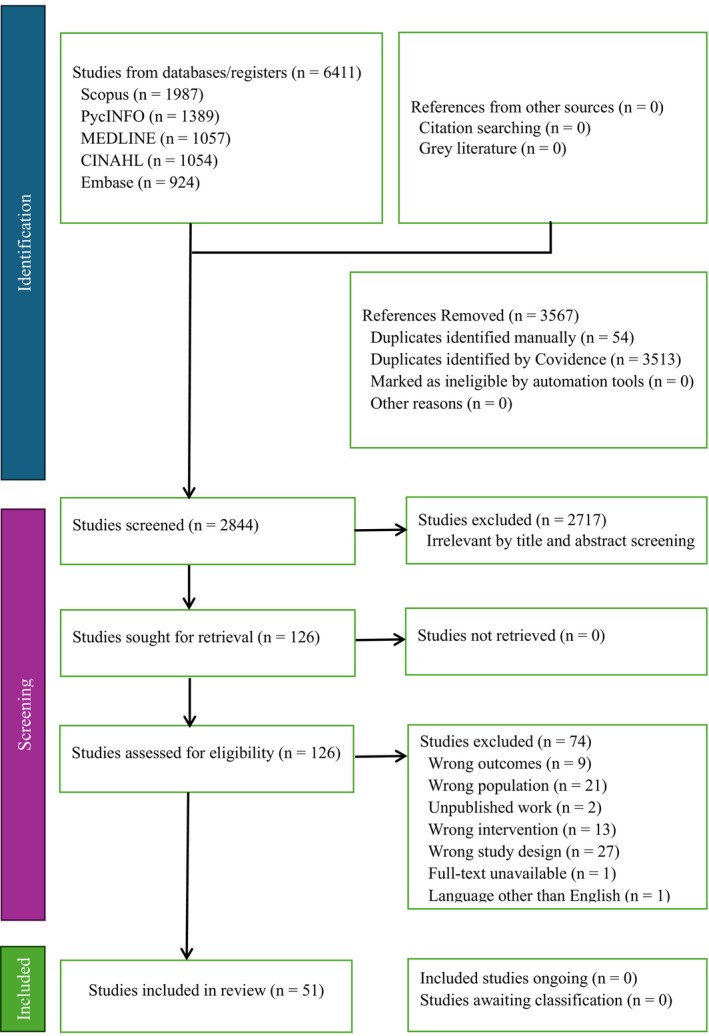
PRISMA flow chart revealing study selection for systematic review and meta‐analysis.

We identified a total of 6411 studies electronically from five databases, removed 3567 references as duplicates, and included 2844 studies in initial screening. We found and removed 2718 studies deemed irrelevant based on the established criteria during the title and abstract screening, and we sought 126 references for full‐text review. We found 51 papers that fulfilled the eligibility requirements from the 126 references retrieved for full‐text review and included them in the synthesis. Figure 1 illustrates the PRISMA flow chart of the study selection for the systematic review and meta‐analysis.

### 3.2 Characteristics of the Included Studies

Table 1 shows the characteristics of the studies, including measured outcomes and results of the individual studies. Among the included studies, 23 (45.1%) were RCT [11, 27, 34, 37, 71, 72, 73, 74, 75, 76, 77, 78, 79, 80, 81, 82, 83, 84, 85, 86, 87, 88, 89], 14 (27.5%) were non‐NRSI [9, 12, 90, 91, 92, 93, 94, 95, 96, 97, 98, 99, 100, 101], and 14 (27.4%) were cRCT [8, 10, 39, 102, 103, 104, 105, 106, 107, 108, 109, 110, 111, 112]. Most of the studies (*n* = 35, 68.6%) were carried out in HIC, of which 31(88.6%) were in the US, and only 16 (31.4%) were conducted in low‐ and middle‐income countries (LMICs). Most of the studies (*n* = 34, 66.7%) targeted parents regardless of sex, mostly with a higher proportion of mothers, whereas 17 (33.3%) exclusively targeted mothers. In 18 (35.3%) studies, comparators did not receive any standard activities, while comparators in 23 (45.1%) studies received an alternative intervention such as general health information. Eight (15.8%) studies measured the outcomes only once after intervention. Thirty‐seven (72.5%) papers stated the application of theory/model in the intervention, with 21 (56.8%) using a single theory or model and 7 (18.9%) using a combination of three or more theories.

**TABLE 1:** Descriptive characteristics of the studies.

Study ID	Design	Trial setting	Country	Country economic class	Target population	Baseline parent	Mother/female guardian/caregiver (%)	Baseline–adolescent	Adolescent female gender (%)	Theory/model used	Method/mode of delivery	Description of the intervention and dose	Time of measurement	Outcome and effects reported
Ahari 2022	cRCT	School‐based	Iran	LMIC	Parent–male adolescent dyads	102	78.72%	102	0	No	Lecture; group discussion; role play; written materials (in hard/soft copies); other: creating scenarios	An intervention designed for four 2‐h sessions that were delivered once per week.	Baseline 4 weeks 3 months	Improved parent‐reported communication about sex‐related topics—baseline/3‐month follow‐up mean difference (MD) is −5.67, *p* < 0.001. Improved parental self‐efficacy in sexual communication—baseline/three‐month follow‐up MD is −3.45, *p* < 0.001.
Aronowitz 2015	NRSI	Organization‐based	US	HIC	Mother–daughter dyads	20	100%	20	20 (100%)	Other: conceptual–theoretical–empirical model (C‐T‐E)	Written materials (in hard/soft copies); other: training: verbal, visual, and experiential as well as repeated exposure to the important constructs (information, motivation, behavioral skills) in each session.	Unspecified	Baseline Post	Mothers report sexual communication mean score is increased by 8.5%. Daughter reported mean sexual communication increased by 9.8% Mean parental confidence to talk (self‐efficacy) increased by 14.50% Adolescent sexual knowledge mean score is increased by 15%
Aventin 2020	cRCT	School‐based	UK	HIC	Parent/guardian/caregiver–adolescent dyads	4097	NA	3337	2131/4097 (52%)	Other: If I were Jack theory of change	Lecture; audio–visual materials; other: an optional homework exercise that invites students to interview their parents/primary caregivers (or another trusted adult such as an older sibling or relative) at home, about their thoughts on Jack and Emma's situation, after they have watched an excerpt of the If I Were Jack interactive film.	The intervention consisted of a 90‐s “hook” feature followed by an 11‐min “instructional” feature, and an optional homework exercise that invites students to “interview” their parents/	NA	Qualitatively showed that misconceptions about discussing sex, religious and cultural influences, lack of sexual health knowledge, and parental unawareness of their role were barriers to while, early and sustained interventions focusing on parents, along with comprehensive sexual and reproductive health education, can help facilitate parental engagement in adolescent sexual health education.
Barker 2019	RCT	Organization‐based	US	HIC	Parent/guardian/caregiver–adolescent dyads	721	57%	721	NA	Social–personal framework	Other: workshop	Interventions delivered over an 8 h workshop to groups of 4–8 individuals/dyads plus a 1 h individual booster session at 2 weeks and a 2 h group booster session 3 months following the workshop.	Baseline 2 weeks 3 months 6 months 12 months	Improved parent‐reported sexual communication, Chen's *d* is 0.28, *p* = 0.05. Adolescent sexual communication, Chen's *d* is 0.21, *p* > 0.05. Multiple sexual partners (2+ partners)—no statistically significant difference, risk difference = 0, *p* > 0.05.
Bartlett 2018	NRSI	Community‐based; School‐based	US	HIC	Mother–daughter dyads	15	100%	15	15 (100%)	No	Lecture; demonstration	Three 1‐h education sessions were conducted by the intervention RN with each mother at a place of the mother's choice. Girl Participants' Group Education consisted of a 2‐h session each week (for 6 weeks) followed by 2 h per week over 6 weeks of service‐learning component of the intervention.	Baseline Post 3 months	Adolescent‐reported mother‐teen sexual risk communication—time one compared against time three is improved, mean difference is 5.17, *p* < 0.03. Adolescent consistent condom use—improved, mean difference for time one compared with time three is 2.07, *p* < 0.03.
Berglas 2016	cRCT	School‐based	US	HIC	Parent/guardian/caregiver–adolescent dyads	301	NA	972	1909 (51%)	Other: Ecological approach	Lecture	Parent education Workshops were offered to all parents of students attending participating schools on a 1‐h orientation and five 1‐h sessions for parents. Sixth sessions, bringing parents and students together, were delivered if the school's schedule permitted. The adolescent component includes a 12‐session curriculum for ninth‐grade students delivered during regular class periods, weekly meetings of peer advocates, and on‐campus sexual health services offered five times a year to all students.	Baseline 1 year	Multiple sexual partners (% yes)—no statistically significant difference, RR is 0.49, *p* > 0.05
Bogart 2013	RCT	Organization‐based	South Africa	LMIC	Parent/guardian/caregiver–adolescent dyads	66	36%	66	44%	Talking parents, healthy teens (TPHT)—a successful US‐based program that led to improved parent–child communication about sex	Lecture; group discussion; role play; written materials (in hard/soft copies)	The intervention consisted of 5 weekly 2‐h group sessions for parents.	Baseline 1–2 weeks	Improved parent‐reported number of sex and HIV topics discussed, multivariate regressions *b*(SE) is 3.26(1.12), *p* is 0.005. Adolescent reported number of sex and HIV topics discussed ()—no improvement, multivariate regressions *b*(SE) is 1.3 (1.1) *p* is 0.24. Improved parental comfort talking to a child about sex—*b*(SE) is 1.0 (0.4), *p* is *p* = 0.015.
Bourdeau 2021	RCT	Web‐based	US	HIC	Parent/guardian/caregiver–adolescent dyads	411	NA	411	226 (55.30%)	Social learning and communication theories	Written materials (in hard/soft copies); audio–visual materials; other: Parent‐adolescent discussion on the selected topic	A web‐based, self‐paced intervention Smart Choices 4 Teens from 2014 to 2015.	Baseline 6 months 12 months 18 months	Improved adolescent‐reported overall sexual communication frequency, GEE, *b*(SE) is −0.437 (0.069), *p* < 0.001.
Brown 2014	RCT	Organization‐based	US	HIC	Parent/guardian/caregiver–adolescent dyads	721	NA	721	410 (57%)	Social–personal framework	Lecture; group discussion; role play; other: skill practice session	Intervention delivered during an 8‐h workshop at each site in groups of four to eight participants	Baseline 3 months	Parent‐reported sexual communication)‐no statistically significant change, adjusted mean difference is 0.24, *p* is 0.94 Adolescent‐reported sexual communication—statistically significant change, AMD is 17.98, *p* < 0.01. Consistent condom use, past 90 days (3 months)—marginally improved, OR is 1.88, *p* is 0.09. Number of sexual Partners past 90 days (3 months)—no statistically significant difference, RR is 1.32, *p* is 0.14. HIV knowledge (adjusted relative change (3 months)‐improved, AMDs is 18.85, *p* < 0.01.
Brown 2021	cRCT	Community based; school‐based; organization‐based	US	HIC	Parent/guardian/caregiver–adolescent dyads	747	NA	886	513/886 (57.90%)	Social cognitive model	Lecture; demonstration; other: the optional text messages, and post‐workshop booster calls for parents	Used over 5 h of workshop using separate and together sessions. The intervention also includes a POST‐LIFT WORKSHOP of 14 TEXT MESSAGES with conversation starters, family activities, and resources (1 per week for 12 weeks + welcome & goodbye) and a BOOSTER PHONE CALL with adult participant 3–5 weeks after the workshop.	Baseline 3 months 12 months	Improved adolescent‐reported parent–child communication about sexuality and pregnancy prevention scale, MD is +5.4%, *p* value (Huber–White RSE [robust standard errors]) is 0.001.
Chambers 2022	RCT	Community‐based	US	HIC	Parent/guardian/caregiver–adolescent dyads	534	87%	534	381/534 (52.62%)	Other: protection motivation theory	Lecture; demonstration; role play; audio–visual materials, Home‐based visit	Delivered a 90–120 min TA–youth session through a home‐based visit. The youth sessions include RCL and HY programs, each composed of nine lessons, delivered through a mix of peer‐group and TA–youth lessons.	Baseline 3 months 9 months	Parent‐reported actual sexual health communication‐no statistically significant change, AMDis 0.06, the *p* value is 0.58.
Chokprajakchad 2020	RCT	School‐based	Thailand	LMIC	Parent/guardian/caregiver–adolescent dyads	80	NA	80	The majority of students were female	Theory of planned behavior	Group discussion; other: computer game media Kid Think	The parental component includes 8 weeks of three components: 1. Interactive group discussions—two 3‐h in‐person sessions for parents. 2. Weekly information graphic (infographic), semi‐structured discussion and group problem‐solving using the line (group) smartphone application. 3. “Talk to Teen” sexual education application provided information with colorful cartoons, pictures, and exciting video clips. The adolescent component includes a game for 60‐min sessions in Weeks 2 and 4, and interactive 30–60‐min classroom discussions about sex education and the prevention of sexual risk behaviors weekly during weeks 1, 2, 4, and 8.	Baseline Post 8 week	Parent‐report sexual communication behavior with—improved, GEE (*B*) 2.37, *p* is 0.003. Adolescent‐report sexual communication with parents—improved, GEE(*B*) is 3.68, *p* is 0.001. Parental attitudes towards sexual communication—improved, GEE (*B*) 20.29, *p* is 0.001.
Colarossi 2014	RCT	Community‐based	US	HIC	Parent/guardian/caregiver–adolescent dyads	71	90.14%	0	NA	Other: social learning and ecological systems theories	Other: workshops	Four 2 h workshops once a week were delivered.	Baseline 6 months	Sexual communication (parent only)—reported “not significant,” but no statistical data given in the report.
Cupp 2013	RCT	Community‐based	Thailand	LMIC	Mother–adolescent dyads	340	100.00%	340	170/340 (50%)	Health belief model; other: value expectancy theory, social learning theory, theories of socialization, social control, social development and family interaction, social inoculation theory	Written materials (in hard/soft copies); other: phone call	A series of five booklets were distributed sequentially, with telephone support provided weekly (on average) by the health educator.	Baseline 6 months	Parent‐reported parent–child communication frequency—marginally improved, *β* = 0.09, *p* = 0.09. Adolescent‐reported parent–child communication frequency—no statistically significant difference, *β* is −0.01, *p* is 0.80.
Dave 2017	NRSI	Community‐based	US	HIC	Parent/guardian/caregiver–adolescent dyads	248	77.91%	0	NA	Social cognitive model; theory of planned behavior; other: social learning and cognitive behavioral approaches.	Lecture; group discussion; other: game, skill practice (communication in pairs and groups, condom skill)	An 18‐h intervention (a curriculum for youth and one for adults), was implemented in 12 weekly 1.5‐h sessions.	Baseline 9 months	Frequency of parent–teen communication about general sex topics (parent at 9‐month follow‐up)‐improved, adjusted DMD = 3.56, *p* < 0.0002. Communication about general sex topics (parent at 9‐month follow‐up)‐improved, adjusted DMD = 3.55, *p* < 0.0002. Communication about sensitive sex topics improved, adjusted DMD 2.93, *p* = 0.0002. Overall communication about sex (parental at 9‐month follow‐up)‐improved, adjusted DMD = 7.57, *p* < 0.0001. Frequency of parent‐teen communication about sensitive topics—improved, adjusted DMD 2.84, *p* = 0.0009. Attitude towards communication (9 months)—no change, adjusted DMD = 0.42, *p* = 0.308. Self‐efficacy of communication (parent at 9 months)‐improved, adjusted DMD 3.47, *p* = 0.001.
Dinaj‐Koci 2015	cRCT	School‐based	The Bahamas	HIC	Parent/guardian/caregiver–adolescent dyads	1833	87.3%	0	55.40%	Other: protection motivation theory	Lecture; group discussion; audio–visual materials	A 1‐h parenting intervention session plus 40‐min booster sessions reinforcing information about communication, monitoring and condom use through a small‐group discussion consisting of parents and youth at 6 and 12 months later.	Baseline 6 month 12 months	Communication about sex (parent report)‐improved, *F*(1, 1315) = 4.59 *p* < 0.05, *d* = 0.11.
Ford 2019	RCT	Organization‐based	US	HIC	Parent/guardian/caregiver–adolescent dyads	78	93.59%	78	50%	No	Written materials (in hard/soft copies); other: health coach discussion in the clinic lobby during well‐care visit	The intervention included a health coach discussing written materials with parents in the clinic within 2 weeks followed by a brief direct verbal and written endorsement of the intervention from the adolescent's clinician and a 2‐week follow‐up telephone call from the health coach to the parent.	Baseline 4 months	Frequency of sex PAC mean score (parent report)—no statistically significant difference, unadjusted: 2.69 vs. 2.40; *p* = 0.19; adjusted: 2.73 vs. 2.46, *p* value = 0.18. Adolescent‐reported frequency of PAC about sex compared with the control group mean score improved‐unadjusted: 2.32 vs. 1.79; *p* = 0.02; adjusted: 2.22 [95% CI, 1.84–2.60] vs. 1.75 [95% CI, 1.45–2.05]; *p* = 0.05.
Grossman 2014	cRCT	School‐based	US	HIC	Parent/guardian/caregiver–adolescent dyads	2018	NA	0	1265/2453 (52%)	Theory of planned behavior	Written materials (in hard/soft copies)	Get real designates parents with family activities (8 in each grade) to give parents (or other caring adults) an opportunity to transmit their values about sex and relationships	Baseline 1 year 3 years	Probability of becoming sexually active for boys (family activities at 7th and 8th grade)—no statistically significant difference, OR = 0.97, 95% CI [0.910, 1.031]. Probability of becoming sexually active for boys (family activities at 6th grade)—statistically significant difference, OR = 0.97, 95% CI [0.934–0.998]
Guilamo‐Ramos 2020	RCT	Organization‐based	US	HIC	Mother–adolescent dyads	900	100%	900	56.44%	No	Lecture; written materials (in hard/soft copies); Other: booster phone call	45 to 60 min of intervention includes a face‐to‐face session, written intervention materials (a family workbook), an HCP endorsement of the intervention to adolescents and mothers; and 1 phone booster session 1 month after the face‐to‐face intervention.	Baseline 3 months 12 months	Sexual debut during past 12 months‐statistically significant difference, RR = 3.12, *p* < 0.05
Hadley 2016	RCT	Organization‐based	US	HIC	Parent/guardian/caregiver–adolescent dyads	170	84%	170	52.39%	Other: the social‐personal framework for HIV‐risk behavior, which is consistent with social learning theory	Written materials (in hard/soft copies); audio–visual materials	Caregiver and Adolescent package comprised of three separate but interconnected components: a short film, intervention modules, and workbook were delivered in approximately 3 h of total time, which included DVD viewing time, individual workbook activities, and joint workbook activities.	Baseline Post 3 months	Parent–teen sexual comm. (parent‐reported)‐small effect, *d* = 0.14, *p* > 0.05 Parent–teen sexual comm. (adolescent‐reported)‐small effect, *d* = −0.3, *p* > 0.05 HIV knowledge, small effect, *d* = 0.23, *p* > 0.05.
Hattakitpanichakul 2019	NRSI	School‐based	Thailand	LMIC	Other: parent/guardian/caregiver–daughter dyads	84	NA	84	95%	Theory of planned behavior	Group discussion; role play; written materials (in hard/soft copies); audio–visual materials; other: practical session for parents using sample situation, homework assignment for adolescents to interview parents	The Program consisted of three sessions in the parent program combined with three sessions in the adolescent program made of up 60–90 min per session over 4 weeks.	Baseline 1 month—female students	Sexual communication behavior (parent‐reported)—improved, value of the GEE *β*(SE) = 2.84(1.0), *p* = 0.004.
Jemmott 2019	RCT	Community‐based	US	HIC	Mother–son dyad	525	100%	525	0%	Social cognitive model; Theory of Planned Behavior	Group discussion; role play; audio–visual materials; other: brainstorming, games, and homework assignments	Although both mothers and sons completed questionnaires, only mothers received the intervention consisting of 16 1‐h modules, with four modules delivered on four consecutive Saturdays in each of four sessions. In addition, two 3‐h stimulation sessions were implemented 3 and 6 months after the initial intervention sessions.	Baseline Post 3 months 6 months 12 months 18 months	Multiple sexual partners—no statistically significant difference, RR = 0.84, *p* = 0.1
Jemmott 2020	RCT	Organization‐based	US	HIC	Parent/guardian/caregiver–adolescent dyads	406	88.18%	406	341/613 (56%)	Social cognitive model; theory of planned behavior	Lecture; group discussion; role play; audio–visual materials; other: homework exercises, and commitment‐to‐practicing‐abstinence letters were written and reviewed by parents and adolescents during the last intervention session.	The faith‐based and nonfaith‐based abstinence‐only interventions consisted of twelve 1‐h modules implemented over three sessions on three consecutive Saturdays. Each had adolescent‐only, parent‐only, and conjoint adolescent–parent components	Baseline Post 3 months 6 months 12 months	Consistent condom uses in past 3 months (18‐months follow‐up period)‐non‐faith‐based—no statistically significant difference, risk ratio = 0.97, *p* = 0.78. Consistent condom uses in past 3 months (18‐months follow‐up period)‐faith‐based‐no statistically significant change, risk ratio = 0.84, *p* = 0.19 Number of sexual Partners in past 3 months (18‐month follow‐up based)—a statistically significant difference, risk ratio = 0.57, *p* = 0.03 Number of sexual Partners in past 3 months (18‐month follow‐up based)—no statistically significant difference, risk ratio = 0.92, *p* = 0.76
Katahoire 2019	cRCT	School‐based	Uganda	LMIC	Parent/guardian/caregiver–adolescent dyads	1700	61.01%	1700	773/1496 (51.67%)	No	Written materials (in hard/soft copies); other: homework exercise, three workshops for parents	The parent component of the intervention consisted of 1‐day workshops that took place at each of the intervention schools and lasted approximately 4 h. The adolescent component was delivered in a classroom over fourteen 90‐min sessions. In addition, a local NGO offered condom education by working with young people as a 2‐h extra‐curricular activity. The second component of the intervention consisted of homework assignments for each of the 14 lessons delivered in the classroom	Baseline Post	Parent‐reported communication frequency on sex and HIV/AIDS‐related topics—improved, the effect sizes were 0.27 (*t* = 3.566; *p* < 0.001). Adolescent‐reported communication frequency on sex and HIV/AIDS‐related topics—improved, the effect sizes were 0.38 (*t* = 4.915; *p* < 0.001).
Lescano 2020	RCT	Community‐based	US	HIC	Parent/guardian/caregiver–adolescent dyads	227	84.58%	227	118/227 (51.98%)	Other: social‐personal framework	Audio–visual materials; Other: separate workshop, Parent‐adolescent dyad engaging didactics, interactive exercises, videos, and in‐depth discussion	Interventions were held for 7 h on one weekend day. During portions of the 7‐h intervention, groups of teens and caregivers received separate workshops, where they participated in interactive group discussions with their peers.	Baseline 3 months	Parent‐report Miller's sexual communication—negative effect, Morris' *d* = −0.06 Adolescent‐report Miller's sexual communication‐negative impact, Morris' *d* = −0.03 Number of past‐90‐day sexual partners—no statistically significant difference, RR = 0.89, *p* = 0.72 HIV knowledge‐small change, Morris' *d* = 0.15
M 2019	RCT	Web‐based	US	HIC	Parent/guardian/caregiver–adolescent dyads	365	74.57%	365	157/355 (45.11%)	Theory of planned behavior, theory of reasoned action	Written materials (in hard/soft copies); audio–visual materials; other: highly interactive content‐based lessons for parents (e.g., clickable activities, open‐ended text box questions, branching activities)	Self‐paced and self‐directed program for parents/caregivers	Pre‐intervention 2 weeks	Frequency of sexual health discussions (parent‐report)‐no statistically significant difference, *b*(SE) = 0.08(0.07), *p* = 0.27. Frequency of sexual health discussions (adolescent‐report)‐not improved, *b*(SE) = −0.02(0.08), *p* = 0.83. Outcome expectancy‐no statistically significant change, *b*(SE) = 0.01(0.03), *p* = 0.80. Comfort towards sex communication‐no change, *b*(SE) = 0.06(0.07), *p* = 0.41. Self‐efficacy‐no change, *b*(SE) = 0.07(0.07), *p* = 0.32.
Majdpour 2021	NRSI	School‐based	Iran	LMIC	Mother–daughter dyads	140	100%	140	140/140 (100%)	No	Lecture; written materials (in hard/soft copies); other: interactive question and answer	The mothers were provided with educational information during three 2‐h sessions in groups.	Baseline 3 months	Attitude towards sex education—improved, mean before = 46.65 vs. mean after = 48.57, *p* < 0.001 for the paired t‐test.
Murry 2014	cRCT	Community based	Georgia	LMIC	Mother–adolescent dyads	670	NA	0	54%	Other: social learning theory, problem behavior theory, and various sociological accounts of delinquency	Lecture; other: game	Seven family meetings were held weekly at local community centers in which youth and caregivers attended separate 1‐h sessions, followed by a 1‐h joint session.	Baseline 3 months 29 months 65 months	Discussions about sexuality (indirect relationship with SAAF) (parent‐reported)—not a statistically significant change, *b* = 0.5, *p* > 0.05.
Murry 2019	RCT	Community based; school‐based; web‐based	US	HIC	Parent/guardian/caregiver–adolescent dyads	418	84%	418	NA	No	Role play; other: guided discussion	The program was delivered over 6 weekly sessions for both the group and technology conditions. For the technology condition, each concurrent parent/youth individual session and family session lasted 45 min on average, resulting in 1.5 h per session, and 9 h of total dosage. For the group condition, each parent/youth concurrent session and family session lasted 1 h on average, resulting in 2 h per session, and 12 h of total dosage.	Baseline Post	Sexual communication (indirect relationship with technology‐based education)‐statistically significant change, *b* = 0.31, *p* value = 0.01; direct relationship with challenging topics, *b* = 0.3, *p* = 0.001, but no statistically significant relationship with group‐based education, *b* = 0.08, *p* > 0.05. Frequency of sexual communication (indirect relationship with group‐based education)‐statistically significant change, *b* = 0.12, *p* value < 0.05; direct relationship with supportive parenting, *b* = 0.78, *p* = 0.001, but no statistically significant relationship with technology‐based education, *b* = 0.01, *p* > 0.05.
Noone 2015	NRSI	Community‐based	US	HIC	Parent/guardian/caregiver–adolescent dyads	59	72.88%	NA	NA	No	Other: interactive theater	A 2‐h theater intervention for parents/caregivers	Pre‐intervention 3 months	Amount of sexual communication (parent only)‐improved, *t* = 3.9707, *p* = 0.0002. Comfort with sexual communication—improved, *t* = 3.2517, *p* = 0.002 for *t*‐test
O'Donnell 2017	cRCT	School‐based	US	HIC	Parent/guardian/caregiver–adolescent dyads	2621	NA	2621	1379/2621 (52.6%)	Innovation diffusion	Audio materials	Intervention sets of seven audio CDs, intended for delivery to families every 4–6 weeks over a school year.	Baseline 3 months 12 months	Sexual initiation—a statistically significant difference (Salud 100 vs. control), AOR = 0.74, *p* < 0.001
Parker 2020	NRSI	Community‐based	US	HIC	Mother–adolescent	41	100%	NA	NA	No	Face‐to‐face session	Included two to three face‐to‐face parent‐promotora sessions lasting from one to 2 h with additional follow‐up phone calls.	Pre‐intervention Post	Parent–child communication about sex (parent only)‐improved, *t*(35) = −4.25, *p* < 0.05. Comfort regarding communication with adolescents about sex—improved, *t*(34) = 2.75, *p* < 0.05
Powwattana 2018	cRCT	Community‐based	Thailand	LMIC	Mother–daughter dyads	78	100%	79	79/79 (100%)	Other: theory of gender and power	Lecture; group discussion; role play; other: role‐specific break‐out sessions, small group activities, sharing experiences, practical communication, mother‐daughter role‐playing, individual practice for self‐risk assessment for daughters, group work, individuals demonstrating verbal persuasion	The parent/caregivers' component includes 3 h weekly intervention administered in 6 weeks. The adolescent component includes “Breaking the voice” is a 3 h weekly intervention administered in 6 weeks, with an introduction and three educational modules per session.	Baseline Post	Sexual communication (parent report)‐improved, *t* = 1.99, *p* = 0.025 Sexual communication (adolescent report)‐not improved, *t* = 0.18, *p* = 0.43 for *t*‐test Attitudes towards sexual communication—no change, *t* = 1.57, *p* = 0.12 for *t*‐test
Promneramit 2021	NRSI	School‐based	Thailand	LMIC	Mother–daughter dyads	79	100%	79	79/79 (100%)	Theory of planned behavior	Use of the computer program to present pictures, graphics, animation, and sound	The intervention includes 6‐h sessions at school, study from the computer‐based Program for the mothers to practice with their daughters at home, and consultation and advice on phone calls.	Baseline Post 1 month	Sexual communication behavior (parent only)‐marginally improved, GEE *b*(SE) = 4.14(2.47), *p* = 0.09. Parental attitudes towards communication‐improved, GEE *b*(SE) = 4.62(1.87), *p* = 0.01.
Puffer 2016	cRCT	Organization‐based	Kenya	LMIC	Parent/guardian/caregiver–adolescent dyads	211	59.61%	237	123/237 (51.90%)	Social cognitive model; Other: ecological transactional theory	Role play; other: family communication practice	The session consisted of a 2‐h program (1‐h family session followed by 2‐h youth meeting) in gender‐segregated groups for discussion and skills practice (e.g., for condom use). Caregivers met together for 30 min to discuss applications of material to marital relationships and parenting and split into male and female discussion groups for the final 30 min. In addition, weekly discussion groups were held for church leaders that focused on identifying ways for church leaders to provide teaching and support to families related to the intervention topics both during and after READY.	Baseline 1 month 3 months	Freq. of communication about sex (3‐month female parent report)‐significant effect, *b*(SE) = 0.71(0.14), *p* < 0.001, Glass's delta = 0.79. Freq. of communication about sex (3‐month male parent report)—significant effect, *b*(SE) = 0.52(0.20), *p* < 0.01 Glass's delta = 0.62. Freq. of communication about sex (3‐month adolescent reports) significant effect, *b*(SE) = 0.53(0.12), *p* < 0.001, Glass's delta = 0.79.
Rink 2021	cRCT	School‐based	US	HIC	Parent/guardian/caregiver–adolescent dyads	12	67.00%	17	9/17 (52.9%)	Other: ecological system theory	Lecture	During the 9‐week intervention period, the parental component (the Native Voices) was implemented three times at the tribal community school in the early evening and the student component (the Native Stand) was implemented twice a week.	Baseline Post	Qualitatively indicated that the trial intervention enhanced parent–child communication in several ways, such as the parents' comfort level in discussing sexual and reproductive health (SRH) with their child, their awareness of how their upbringing affected their communication about SRH, and their comfort level when seeking SRH services for their child
Romo 2014	NRSI	Community‐based; school‐based	US	HIC	Mother–daughter dyads	48	100%	48	48/48 (100%)	No	Lecture; group discussion; one‐on‐one exercise	The program consisted of three 2‐h sessions spaced 1 week apart. The mothers attended the first session together without their daughters and the other two sessions with their daughters.	Baseline Post	Self‐reported frequency of communication about sexual topics (mother‐reported)‐improved, mothers, *t*(1,45) = 5.25, *p* < 0.001. Self‐reported frequency of communication about sexual topics (daughters)—improved, *t*(1,44) = 4.82, *p* < 0.001. HIV transmission risk knowledge—a statistically significant difference, *t*(1,47) = 6, *p* < 0.001.
Rousta 2019	RCT	School‐based	Iran	LMIC	Mother–son dyad	90	100%	0	NA	No	Lecture; other: question and answer	Intervention was given through 1 h of four training sessions once a week.	Pre‐intervention 2 weeks after intervention	Attitude score End of study‐improved, for Man‐Whitney U test result (*z* = 7.55), *p* < 0.001.
SantaMaria 2018	RCT	Organization‐based	US	HIC	Parent/guardian/caregiver–adolescent dyads	39	79%	0	NA	No	Lecture; written materials (in hard/soft copies)	A 30‐min face‐to‐face orientation session on handouts and manual, which the parent then took home to use with their child.	Baseline 1 month	Frequency of communication past 3 months (Parents only)‐improved, *t* = −2.13, *p* = 0.03.
SantaMaria 2021	RCT	School‐based	US	HIC	Parent/guardian/caregiver–adolescent dyads	519	90.27%	508	255 (50.58%)	Theory of planned behavior	Written materials (in hard/soft copies); Other: The face‐to‐face session, booster call	Forty‐five minutes of face‐to‐face session with the parents to review the FTT + HPV materials, motivate parents to talk with their children, and address specific components of the program. Parents received a manual as well as three handouts to supplement the face‐to‐face session.	Baseline 1 month 6 months	Frequency of communication (parent‐report)‐improved, *p* = 0.001. Frequency of communication (adolescent report)‐not improved, *p* = 0.19. Parent communication expectancy (6 months)—not improved, *p* = 0.85. Parent communication self‐efficacy (at 6‐month follow‐up)‐not improved, *p* = 0.21. Youth condom knowledge—Improved, *p* = 0.04. Youth HIV/STI knowledge—not improved, *p* = 0.13.
Seif 2019	NRSI	Community‐based	Tanzania	LMIC	Parent/guardian/caregiver–adolescent dyads	1000	66.70%	0	NA	Other: information‐motivation‐behavioral skills (IMB)	Lecture; group discussion; role play; game, brainstorming	A total of over 10 h of sessions were held with separate and together sessions for parents/caregivers and adolescents. The intervention is carried out for two conservative days and 5 h and 25 min of training in each session.	Baseline Post 1‐month 6 months 1‐year	Communication (parent‐reported at 1‐year follow‐up)—improved, *F*(1,827) = 40.44, (*p* < 0.001), *d* = 0.4. Parental attitude—improved, *F*(1,827) = 49.4, (*p* < 0.001), *d* = 0.3. Parental communication behavioral skill self‐efficacy—no statistically significant change, *F* = 0.95, *p* = 0.33, *d* = 0.07.
Shegog 2021	NRSI	Web‐based	US	HIC	Parent/guardian/caregiver–adolescent dyads	10	80%	10	3/10 (30%)	Social cognitive model; PRECED–PROCEED; other: social influence models, and the theory of triadic influence	Internet‐based game	Self‐paced game on cell phone	Baseline 2 weeks	Communication about sex outcome expectation (parents at 2 weeks follow‐up)‐improved, Wilcoxon signed rank (MD) *p* = 0.03 Communication about sex outcome expectation (adolescents at 2 weeks follow‐up)—not improved, Wilcoxon signed rank (MD) *p* = 0.99 Communication about sex self‐efficacy (parents at 2 weeks follow‐up)‐not improved, Wilcoxon signed rank (MD) *p* = 0.32
Stanton 2015	cRCT	School‐based	Bahamas	HIC	Parent/guardian/caregiver–adolescent dyads	NA	NA	1436	821/1436 (57.17%)	Social cognitive model; other: protection motivation theory, decision‐making model (stop, options, decision, action [SODA])	Group discussion; demonstration; audio–visual materials; other: Parent‐adolescent discussion	BFOOY include games, interactive discussions, role plays, and homework exercises to reinforce main messages and to increase knowledge and skills regarding risk avoidance. CImPACT includes a 22‐min video followed by a discussion between the parent and youth. GFI is also a 22‐min video.	Baseline 6 months 12 months 18 months	Consistent condom use—improved, AOR = 3.85, *p* = 0.001 HIV/AIDS knowledge (at 18‐month follow‐up, HELE is the reference)—improved effect, *t* = −3.29, *p* < 0.001
Stanton 2016	cRCT	School‐based	The Bahamas	HIC	Parent/guardian/caregiver–adolescent dyads	NA	NA	1436	607/1223 (49.63%)	Other: protection motivation theory	Group discussion; role play; audio–visual materials; other: games, practice on condom use	The parental component, CImPACT, includes a 22‐min video illustrating parent–child conversation about sex. Following the video, the parent and child engage in role‐play and discussion and conclude with the youth and parent practicing the correct condom use.	Baseline 6 months 12 months 18 months 24 months	Parent‐adolescent communication (adolescent report)‐no statistically significant change controlling for prior FOY exposure, *b*(SE) = 0.02 (0.07), *t* = 0.24, *p* > 0.05. Consistent condom uses at 24‐month follow‐up—improved, *b*(SE) = 0.87 (0.34), *t* = 2.56, *p* < 0.05 HIV/AIDS knowledge—no statistically significant difference controlling for prior FOY exposure, *b*(SE) = 0.00 (0.01), *t* = 0.61, *p* > 0.05
Tarantino 2014	RCT	Community‐based	South Africa	LMIC	Mother–adolescent dyads	99	100%	99	52 (53%)	Social cognitive model; theory of reasoned action; other: social learning theory, problem behavior theory	Lecture; group discussion; role play; other: homework, modeling, and skill practice	Imbadu Ekhaya (parent‐based HIV prevention intervention) delivered over six 2.5‐h‐long sessions with groups of female caregivers and their adolescent children (child included in Session 6 only).	Baseline, 1 month 6 months	Communication about sex with intimate partner violence (IPV) as mediator variable (*p*)—IPV significantly improved the effect on communication, *B* = −4.27, *p* < 0.05. Communication about sex with neighborhood safety (safety) as mediator variable (*p*)‐with a neighbor safety as a mediator variable, statistically significant effect on communication about sex, *B* = 1.17, *p* < 0.01. Communication about sex with decision‐making power (DM) as mediator variable (caregiver report at 6 months)—improved, *B* = −4.6, *p* = 0.07.
Tarantino 2016	NRSI	Organization‐based	US	HIC	Mother–adolescent dyads	12	100%	12	NA	Theory of planned behavior; other: attachment theory	Lecture; role play; audio–visual materials; discussions, games, homework, home visit	Three 4‐h weekly mothers' group sessions were held with an additional 1.5‐h family home visit with a facilitator within 1–3 weeks following the last group session.	Baseline Post	Communication frequency about sex (parent‐reported)‐no difference, very small effect size, *t* = −0.37, *p* = 0.72, *d* = 0.09. Communication frequency about sex (Adolescent)—small effect size, *t* = −1.6, *p* = 0.14, *d* = 0.22.
Thurman 2018	NRSI	Community‐based	South Africa	LMIC	Mother–adolescent dyads	178	100%	216	64/105 (60.95)	No	Lecture	Let's Talk program was held with 19 caregivers and 14 adolescent sessions, of which six were joint caregiver‐adolescent sessions. Participants meet in small closed‐group 90‐min sessions led by a trained facilitator and cover predetermined topics in sequence	Baseline 3 months	Caregiver sexual communication (adolescent only)—improved, GEE coefficient = 1.745, *p* < 0.0001, 20.922% mean change. HIV transmission knowledge—statistically significant change, GEE coefficient = 0.417, *p* = 0.008, % mean change = 7.31. Condom knowledge—statistically significant GEE coefficient = 0.586, *p* < 0.001, mean change = 17.33%.
Varas‐DÃaz 2019	RCT	Web‐based	US	HIC	Parent/guardian/caregiver–adolescent dyads	660	89.55%	660	342/660 (51.82)	Social cognitive model; Theory of planned behavior; theory of reasoned action; other: eco developmental theory	Audio–visual materials; other: animation, interactivity	The web‐based program allowed the parents to access the program for 3 months. Parents were directed to a “homework” activity to be completed with their adolescents on completion of the web‐based modules.	Baseline 3 months 6 months 12 months	Sexual peer pressure communication (parent‐reported)‐improved, mean difference = 0.01, standard error = 0.01, *p* = 0.03. Sexual prevention communication (parent‐reported)‐improved, mean difference = 0.01, standard error = 0.01, *p* = 0.03. Sexual protection communication (parent‐reported)‐improved mean difference = 0.02, standard error = 0.01, *p* = 0.007. Sexual risk communication (parent‐reported)‐improved, mean difference = 0.01, standard error = 0.01, *p* = 0.009.
Wang 2014	cRCT	School‐based	US	HIC	Parent/guardian/caregiver–adolescent dyads	894	NA	1233	1447 (56.4%)	Social cognitive model; other: protection motivation theory	Role play; other: interactive discussion, games, homework exercises, fictional family stories.	The youth experimental condition was BFOOY which contains 10 sessions that include interactive discussions, role‐plays, games, and homework exercises to reinforce main messages and increase knowledge and skills regarding risk avoidance.	Baseline 6 months 12 months 18 months	Parent‐adolescent communication (adolescent only)—improved overall effect, BFOOY + CImPACT compared to BFOOY only (ref), *b*(SE) = 0.24 (0.07), *t* = 3.12, *p* < 0.01.
Zhang 2018	RCT	Community‐based	US	HIC	Mother–son dyads	525	100%	525	0	Social cognitive model; theory of planned behavior	Group discussion; role play; audio–visual materials; other: brainstorming, music, games, homework assignment	The intervention was highly structured and consisted of 16 1‐h modules, with four modules delivered during each of the four sessions on four consecutive Saturdays. In addition, two 3‐h booster sessions were implemented 3 and 6 months after the initial intervention sessions.	Baseline 3 months 6 months 12 months 18 months	Communication about sexual health (parent only)‐improved, GEE coefficient *b* = 0.22, *p* = 0.006.
Ziaei 2020	RCT	Community‐based	Iran	LMIC	Mother–daughter dyads	168	100%	168	84/84 (100%)	No	Other: receiving feedback, summarizing the previous session and reviewing the assignments, running scenarios designed in small groups and presenting it to a large group, mothers explaining their experiences of communication skills with their daughters, the conclusion of topics by a counselor at the end of each session and presenting homework assignments	The mothers' component consisted of consultation in groups for 6–7 weekly sessions, each one lasting 60 min.	Baseline 1 week 3 months	Mother‐daughter sex dialogue from the mothers' viewpoint (parent‐reported)—statistically significant difference between the mothers in the intervention and control groups in terms of their average score of sex dialogue intervention: 30.41, control: 42.47, *p* = 0.009.

The included interventions encompassed a variety of structured educational sessions aimed at both parents and adolescents, focusing on enhancing communication and knowledge regarding sexual health and risk avoidance. The approach included group workshops, individual sessions, educational materials, discussions, activities, booster sessions, and follow‐up activities to reinforce learning and engagement over an extended period. The interventions range from 1‐h modules to 8‐h workshops, with some including weekly sessions over several weeks to months. The delivery methods vary, including in‐person sessions, phone calls, text messages, and web‐based platforms. The time frame for conducting the last outcome measurement ranges from 0 to 65 months after the intervention, with a mean and standard deviation (SD) of 8.5 and 11.5 months, respectively (see Table 1).

### 3.3 Parent‐Related Sexual Health Communication Outcomes

#### 3.3.1 Sexual Health Communication Frequency

Of the total 51 studies included in the synthesis, 39 (76.5%) studies [8, 9, 11, 12, 27, 34, 37, 71, 72, 73, 74, 75, 77, 80, 81, 83, 84, 85, 86, 87, 88, 89, 90, 91, 93, 94, 95, 96, 97, 99, 100, 102, 103, 105, 106, 108, 109, 111, 112] assessed change in parent–adolescent SRH communication. Thirty‐three (84.6%) of the 39 studies [8, 9, 11, 12, 27, 37, 71, 72, 73, 74, 75, 77, 80, 81, 83, 84, 85, 86, 87, 88, 89, 91, 93, 94, 95, 96, 97, 99, 103, 105, 106, 108, 109] assessed the relationship between parent‐reported communication on SRH topics, from which 23 (70.6%) reported a statistically significant change [8, 9, 11, 12, 71, 73, 77, 83, 84, 85, 86, 87, 88, 89, 91, 93, 94, 96, 97, 103, 105, 108, 109]. Two (5.9%) studies [75, 95] reported marginally improved communication frequency, *β* = 0.09, *p* = 0.09, and GEE *b*(SE) = 4.14(2.47), *p* = 0.09, respectively. In comparison, 9 (26.5%) studies reported no statistically significant change [27, 37, 72, 74, 77, 80, 81, 99, 106].

Furthermore, out of the 39 studies, 22 (56.4%) evaluated how frequently adolescents communicated about their SRH. Of these, 14 (63.6%) found statistically significant changes [9, 27, 34, 37, 73, 90, 96, 99, 100, 102, 105, 108, 109, 112]; the other eight studies [11, 71, 75, 77, 80, 81, 83, 111] showed no statistically significant improvement as reported by adolescents.

In 16 (41.0%) of the 39 studies, the frequency of communication about SRH was looked at from both parents' and adolescents' perspectives [9, 11, 27, 37, 71, 73, 75, 77, 80, 81, 83, 96, 99, 105, 108, 109]. Of these, 6 (37.5%) studies showed an increase in communication frequency as reported by both parents and adolescents [9, 73, 96, 105, 108, 109], whereas others found no difference [80, 81]. However, although parents reported a significant change in sexual health communication after the intervention, there was no significant difference in communication frequency reported by adolescents [11, 71, 77, 83] in the same study and vice versa [27, 37, 99], indicating a discrepancy in the perceived effectiveness of the intervention between parents and adolescents in some studies.

**FIGURE 2:**
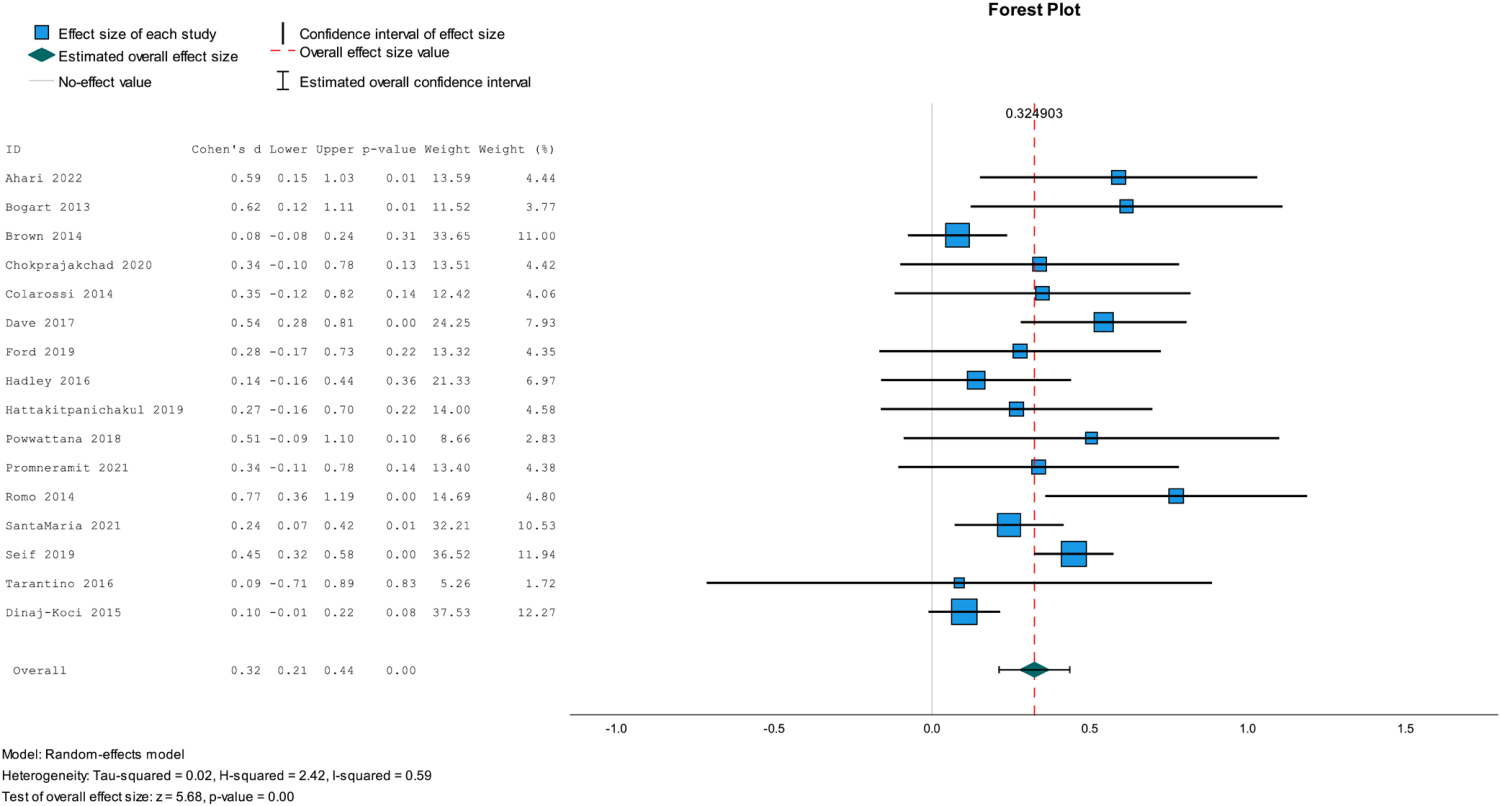
Meta‐analysis of the overall studies on parent‐reported parent‐adolescent sexual health communications.

We included studies with the required data from RCTs, cRCTs, and NRSIs in the meta‐analysis to determine the overall impact size for parent‐reported sexual health communication frequency. The analysis demonstrated a small effect size (Cohen's *d*) of 0.32; the *I* ^2^ test of heterogeneity was 59% (see Figure 2). The subgroup meta‐analysis findings suggested that interventions conducted in community, school, and organization‐based settings, defined as physical workplaces, community‐based organizations, health care settings, and others, have varying effects on parent‐reported sexual health communication, *d* = 0.48, 0.24, and 0.16, respectively. Moreover, different effect sizes were observed in low‐ and high‐income countries (HICs) regarding parent‐reported communication frequency, *d* = 0.56 and 0.26, respectively, with a reporting bias found in low‐income countries, ˆβ0E = 0.45, *p* < 0.01. Furthermore, the effect sizes across different study designs were small, *d* = 0.21, 0.33, and 0.46, respectively. The predicted intercept value, ˆβ0E, for NRSI was 0.49 (*p* = 0.01), based on an Egger's regression‐based asymmetry test.

Our review of two included qualitative studies showed the effectiveness of parent‐based sexual health education interventions and potential barriers. One of the studies showed that misconceptions about discussing sex, religious and cultural influences, and parental unawareness of their role were barriers to parental engagement in adolescent sexual health education, whereas early and sustained interventions focusing on parents, along with comprehensive sexual health education intervention, can help facilitate parental engagement in this important aspect of adolescent development [10]. The pilot intervention resulted in improved communication among parents, including feeling more comfortable discussing SRH with their child, increased communication within the parent dyad about SRH topics, awareness of how their upbringing influenced their communication about SRH, and increased comfort in seeking SRH services for their child in another qualitative study [101].

In addition, out of the 39 studies, 22 (56.4%) evaluated how frequently adolescents communicated about their SRH. Of these, 14 (63.6%) reported statistically significant improvement in sexual health communication [9, 27, 34, 37, 73, 90, 96, 99, 100, 102, 105, 108, 109, 112]; the other 8 (36.4%) studies [11, 71, 75, 77, 80, 81, 83, 111] showed no statistically significant improvement as reported by adolescents. Two studies [73, 77] that were found to be outliers and contributed 38.82% of the overall heterogeneity and 38.70% of the RCT‐specific heterogeneity were excluded from the meta‐analysis on the frequency of sexual health communications reported by adolescents.

**FIGURE 3:**
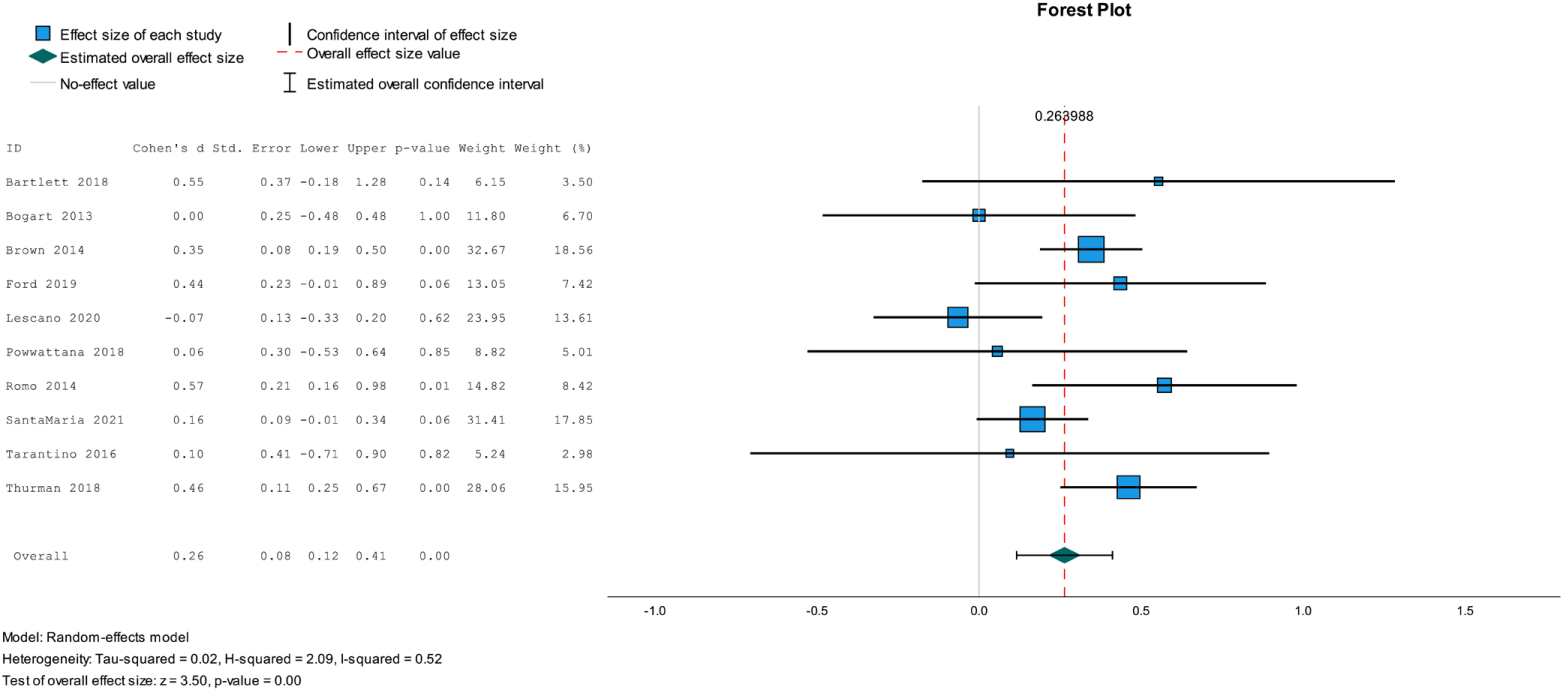
Meta‐analysis of overall studies on adolescent‐reported parent‐adolescent sexual health communication frequency.

Finally, five RCTs [27, 37, 71, 80, 83], four NRSI [90, 96, 99, 100] and one cRCT [108] were incorporated into the meta‐analysis to ascertain the pooled intervention effect on adolescent‐reported parent–adolescent sexual health communication frequency. The combined analysis, independent of the study design, yielded a small effect size, Cohen's *d* = 0.26, and moderate to significant heterogeneity, *I* ^2^ = 52% (see Figure 3). No reporting bias was detected in this test (ˆβ0E = 0.27, *p* = 0.13). Based on a subgroup meta‐analysis of the overall studies on adolescent‐reported communication frequency, studies in HICs and organizational settings had effect sizes of 0.26 and 0.32, respectively. Furthermore, RCT studies showed an effect size of 0.18 with moderate to significant heterogeneity, while NRSI‐based studies had a moderate effect size of 0.47 with no heterogeneity. Additionally, subgroup analysis of the intervention effect based on theory use indicates an effect size of 0.26, with the no‐theory use studies having 0.48.

#### 3.3.2 Parental Attitude Toward Sexual Health Communication

Of the seven studies that assessed parental attitudes toward sexual health communication [12, 73, 82, 92, 95, 97, 108], five studies [73, 82, 92, 95, 97] showed a statistically significant difference.

**FIGURE 4:**
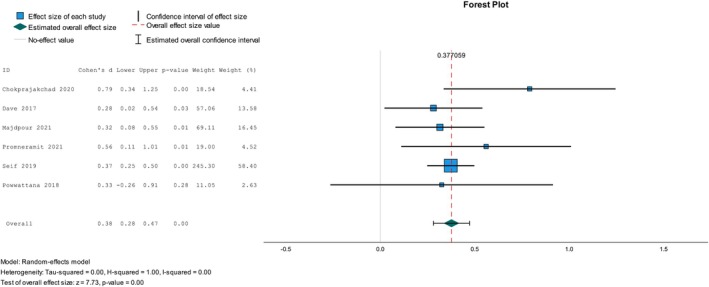
Meta‐analysis of the overall intervention effect on parental attitude toward sexual health communication.

We conducted a meta‐analysis of six studies that comprised four NRSI studies [12, 92, 95, 97], one RCT study [73], and one cRCT study [108] to assess the pooled effect of interventions on parental attitudes towards sexual health communication. We first conducted a meta‐analysis of all six studies, regardless of their research methodologies, to determine the overall intervention impact. We then carried out a separate meta‐analysis for NRSI. The overall analysis showed a small intervention effect size (*d* = 0.38, 95% CI = 0.28, 0.48, *p* < 0.001). The *I* ^2^‐test revealed no heterogeneity in this analysis (see Figure 4); however, Egger's regression‐based asymmetry test indicated the existence of reporting bias; the estimated value of the intercept in Egger regression ˆβ0E was 0.30 (*p* = 0.04). A meta‐analysis that included only NRSI revealed a similarly small effect size on parental attitudes toward sexual health communication (*d* = 0.36, 95% CI = 0.26, 0.46, *p* < 0.001). Neither heterogeneity nor publication bias was observed in this test, *I* ^2^ = 0 and Egger's regression‐based asymmetry test (ˆβ0E = 0.34, *p* = 0.11).

#### 3.3.3 Parental Self‐Efficacy Toward Sexual Health Communication

**FIGURE 5:**
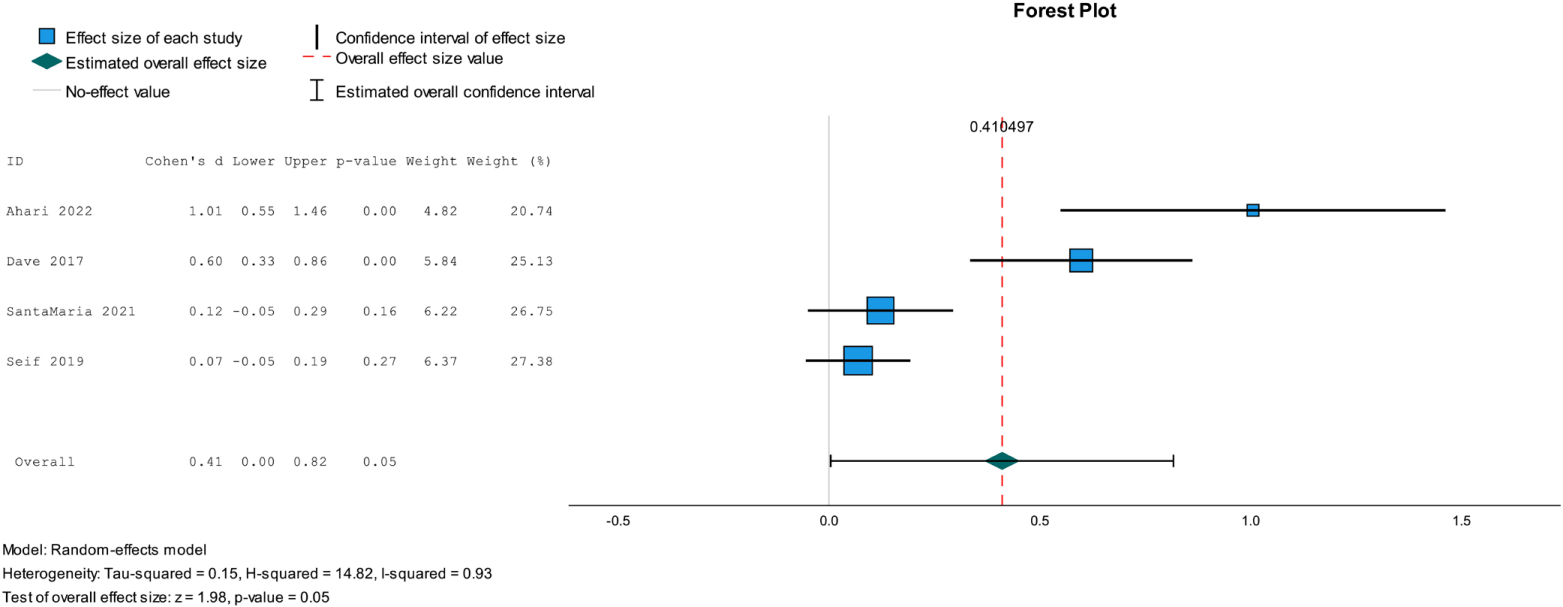
Pooled effect of interventions on parents' self‐efficacy toward sexual health communication with their adolescent children.

Of the six studies [8, 9, 12, 81, 83, 97] that examined parental self‐efficacy in communicating about sexual health, three reported a statistically significant difference [8, 9, 12]. We conducted a meta‐analysis of four studies, including one RCT [83], one cRCT [8], and two NRSIs [12, 97], to determine a pooled effect size on parents' perceived self‐efficacy toward sexual health communication with their adolescent children. Figure 5 shows the pooled effect from these studies (*d* = 0.41, 95% CI = 0.00, 0.82, *p* = 0.05) and the degree of heterogeneity (*I* ^2^ = 93%). Egger's regression‐based test showed no reporting bias in this test (ˆβ0E = −0.33, *p* = 0.10).

#### 3.3.4 Parental Comfort Toward Sexual Health Communication

Only four studies [71, 81, 93, 94] assessed the intervention's effect on parental comfort in sexual health communication with their adolescents. While one was carried out in South Africa [71], the other three were carried out in the US [81, 93, 94]. Two studies [71, 81] were RCTs, whereas the other two studies [93, 94] were NRSI studies. Except for one study [81], the other three studies found a significant change in parental comfort toward sexual health communication with their adolescents.

#### 3.3.5 Parental Outcome Expectation Toward Sexual Health Communication

The effect of parent‐based sexual health education interventions on parental outcome expectancy was surprisingly only evaluated in three studies [81, 83, 98]. All three studies were conducted in the US. One study used NRSI [98], whereas the other two studies were conducted by RCT [81, 83]. There was no significant difference between intervention and control groups in two of these studies [81, 83], whereas the other study reported a statistically significant difference [98]. Because of insufficient studies with raw data, we did not perform a meta‐analysis for this outcome.

### 3.4 Adolescent SRH Behaviors

#### 3.4.1 Multiple Sexual Partners Among Adolescents

Seven studies conducted parent‐based sexual health education interventions [11, 27, 39, 78, 79, 80, 90] aiming to reduce the number of sexual partners, of which six studies [11, 27, 39, 78, 80, 90] found no significant difference. In contrast, one 3‐arm study found a reduced number of sexual partners in the non‐faith‐based education group. At the same time, there was no statistically significant change in the faith‐based education group compared with the control group [79].

**FIGURE 6:**
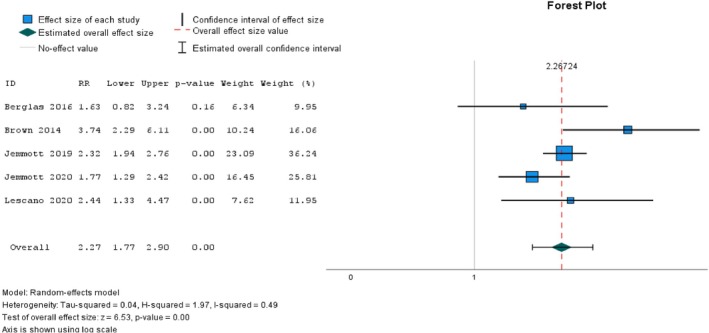
Pooled risk ratio on multiple sexual partners among adolescents.

Another outlier [11] was identified in the meta‐analysis of multiple sexual partners. This study alone accounted for 46.73% of the heterogeneity detected and substantially affected the effect size. Removing the outlier, we considered five studies, four RCTs, and one cRCT, for meta‐analysis. Figure 6 shows the pooled intervention effect on multiple sexual partners among adolescents. A meta‐analysis of six studies—four RCTs [27, 78, 79, 80] and one cRCT [39]—showed a statistically significant risk ratio (RR = 2.27, 95% CI = 1.17, 2.90, *p* < 0.001). There was moderate heterogeneity, with an *I* ^2^ of 49%.

#### 3.4.2 Sexual Debut

**FIGURE 7:**
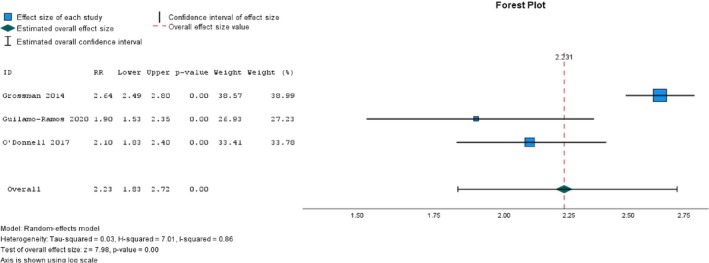
Pooled risk ratio on sexual initiation among adolescents.

Three studies measured sexual debut and found delayed sexual first experiences [76, 104, 107]. All these studies were conducted in HIC; two were conducted in schools, whereas the other was organization‐based. Two studies were cRCT, and the other was RCT. All these studies were included in the meta‐analysis (see Figure 7). We used a precalculated effect size and standard error to determine the pooled intervention effect. The analysis showed a statistically significant pooled log risk ratio (RR = 2.23, 95% CI = 1.83, 2.72, *p* < 0.001). All the studies individually revealed a risk ratio of more than 1 with *p* values less than 0.001 in the analysis. We also found a significant heterogeneity and publication bias with an *I* ^2^ of 86% and Egger's Regression‐based asymmetry test (ˆβ0E = 1.12, *p* = 0.03) in this analysis.

#### 3.4.3 Consistent Condom Usage

Of the five studies assessed, the effect of parent‐based sexual health education interventions on consistent condom use [27, 79, 90, 110, 111] was examined. Three of them found improvement in consistent condom use [90, 110, 111], and one study [27] reported marginally improved use, *p* = 0.09, while the other study [79] reported no difference.

## 4 Discussion

### 4.1 Parent–Adolescent Communication

Our review shows that parent‐based sexual health education interventions are associated with increased parent–adolescent sexual health communication, parental attitudes, and self‐efficacy toward sexual health communication. This was consistent with a finding from another review, which reported increased communication frequencies and parental comfort with communication [20]. However, most of the included studies were conducted in HICs, indicating a meaningful lack of representation from LMICs, given the disproportionately high level of HIV [113] and unwanted pregnancies [40, 114] in these settings. This finding of underrepresentation was consistent with the report from a systematic review conducted globally [23] and in LMICs [25]. Limited research and insufficient implementation of effective parent‐based sexual health education interventions increase the risk of preventable health issues among adolescents due to inadequate communication and education. This could lead to a higher demand for healthcare resources to address these preventable health problems, putting further strain on the healthcare system. In addition, while many studies included mothers as participants in parent–adolescent dyads, there is a lack of research involving fathers. This finding was consistent with the previous report [20, 115]. This may perpetuate the stereotype that sexual health education is primarily a “woman's” job, as demonstrated by other studies [94, 115, 116, 117]. In addition, evidence indicated that mothers worry about possible problems if their husbands discover that their daughters are looking for information about sexuality [92], highlighting cultural and social implications, especially in cultural contexts in which fathers hold a more dominant decision‐making role in the family. In cultural contexts where fathers hold more dominant decision‐making roles in the family, traditional gender roles and expectations are emphasized, leading to a power dynamic where fathers have significant influence over family decisions and the behavior of other family members [118]. The opinions and actions of the father figure are highly regarded and may shape the family's attitudes and behaviors. The meta‐analysis's findings highlight the critical role of both mothers and mixed‐gender parents in improving parent–adolescent communication about sexual health, with mothers having a much greater impact on adolescent‐reported communication. The difference observed in parent‐reported sexual health communication could be explained by the reporting bias in the studies that involved fathers. The partial involvement of parents in these programs has, therefore, neglected the significant role of fathers, which could have a negative impact on the information and support adolescents receive regarding SRH.

Our results have shown that parent‐based sexual health education interventions can lead to improved parent–adolescent sexual health communication. Although the results of the systematic review indicated a mixed effect on communication, in line with other systematic reviews [23, 24, 25], the pooled effect from the meta‐analysis demonstrated a positive effect on the reported frequency of sexual health communication both by the parents and the adolescents, which was also in line with findings from another study [23]. The observed change in sexual health communication could be attributed to a shift in parental attitude and self‐efficacy in our review, which is consistent with other evidence [42, 49]. Raising parents' comfort levels in talking to their children about SRH could be another important factor in facilitating the observed change in parent–adolescent sexual health communication [101]. This suggests that parent‐based sexual health education intervention serves as a tool to help parents comprehend the importance and strategies for communicating with adolescents about their SRH and thereby support adolescents in developing appropriate sexual behavior. This further highlights how important it is to maintain and disseminate these programs to include more communities and areas, especially the LMICs, where HIV and unwanted pregnancies are disproportionately affecting adolescents. This could be supported by evidence from other studies that indicate improved parent–adolescent communication may help reduce health disparities associated with HIV/STIs and may also help prepare adolescents for healthier sexual behavior [9, 41].

The systematic review highlighted that 72.5% of the parent‐based sex education interventions were informed by theoretical frameworks. This indicates that many studies utilized established theories or models to guide the design and implementation of their interventions. The use of theory is critical as it can enhance the effectiveness of the programs by providing a structured approach to understanding and addressing the factors influencing parent–adolescent communication and sexual health outcomes. However, despite the prevalence of theoretical frameworks, our review noted positive effects in the theory use but a smaller effect size than that of non‐use studies, both in parent‐reported and adolescent‐reported sexual communication frequencies, suggesting that the application of these theories might not uniformly translate into successful outcomes. Further research is necessary to explore how different theories impact the effectiveness of parent‐based interventions in different contexts.

### 4.2 Parental Attitude Toward Sexual Health Communication

Our review demonstrated that parent‐based sexual health education interventions have a promising effect on parental attitudes toward sexual health communication. Most of the identified studies showed improved parental attitudes over time. This may be supported by the evidence that a change in attitude takes more time to be seen [108]. Positive attitudes tend to facilitate better sexual health communication between parents and adolescents. For instance, data from certain studies indicate a significant relationship between parents' attitudes and the extent of their discussions about sexual and reproductive issues with adolescents [25, 49].

However, our review also showed that mothers continue to hold the belief that their daughters' understanding of sexuality and contraception could encourage them to engage in extramarital affairs, highlighting that they should not receive sexual education until after marriage [92]. This finding was consistent with a finding from a systematic review conducted in the United States of America [42]. This could be a major barrier in parent‐based sexual health education interventions. This may be due to differences like the nature of the messages in the education, the contexts in which the messages were delivered, the recipients, the source, the follow‐up period, and others.

### 4.3 Parental Self‐Efficacy Toward Sexual Health Communication

According to our review, the systematic review indicated varying effects of the parent‐based sexual health education interventions on parents' self‐efficacy in communicating about sexual health. This was in line with a finding from a previous review [42]. The effect in the meta‐analysis, however, indicated that the intervention has a positive effect on parents' self‐efficacy in sexual health communication. This finding aligned with another review report, where parents reported increased self‐efficacy in their communication skills [42].

Self‐efficacy, as defined in the introduction, is one of the central determinants of “behavior” [119]. Albert Bandura hypothesized that expectations of personal efficacy support coping behavior in the face of difficulties and unpleasant experiences. This theory also influences the effort put forth, whether coping behavior is initiated, and how long it is sustained [120]. As such, the result from the current study suggests that parent–adolescent sexual health communication may benefit from interventions that enhance parental self‐efficacy. More research is also needed to find specific evidence for practice and policy.

### 4.4 Adolescent SRH Behaviors

The systematic review indicated inconsistent changes in condom use and no difference in the number of multiple sexual partners among adolescents. However, the meta‐analysis of five studies (four RCTs and one cRCT) suggests that adolescents in the intervention group were more than twice as likely to have multiple sexual partners compared to those in the control group. These studies were conducted in the US. Without protection, multiple sexual partners increase the risk of HIV or other STIs [121], unintended pregnancies, and repeat abortions [122, 123]. Our study did not assess the links between multiple sexual partners and increased risks of STIs or pregnancy. Further research is needed to explore how intervention programs may have contributed to increased sexual partners and their potential risks. Despite an extensive review, no study has specifically examined the impact of these interventions on sexual debut, unintended pregnancy, and abortion. This gap highlights the need for further research into the long‐term effects of parent‐based sex education on teenage pregnancy, abortion outcomes, and overall adolescent reproductive health.

### 4.5 Limitations

This review synthesized evidence from various studies on parent‐based sexual health education interventions, marking the first global analysis of such interventions across different social, cultural, and economic contexts. However, there are notable limitations, and care should be taken in the interpretation of the findings. First, a high risk of bias was identified in this review. The risk of bias was assessed at the outcome level, and almost all outcomes demonstrated a high risk. Incomplete information was the primary cause of the assessed potential threat of bias, suggesting future research should utilize diverse data sources, including registered protocols and raw data, for better quality. Second, the heterogeneity in study results may stem from variations in populations, cultural contexts, intervention types, and follow‐up durations [20]. We observed variations in specific parent‐based sexual health education interventions conducted in settings such as low‐ and high‐income countries, including separate analyses for study designs. These analyses have also revealed higher levels of variation. The random‐effects model was employed to account for both within‐study error and between‐study variability, allowing for flexibility in treating each study as estimating its effect. This variability highlights the need for standardized procedures in parent‐based sexual health education interventions to develop consistent guidelines and resources. At last, the focus on peer‐reviewed English‐language journals may have excluded significant studies, and the exclusion of certain outlier studies may have influenced the meta‐analysis results. However, conducting analyses both with and without outliers provides a more nuanced understanding of the findings.

## 5 Conclusion

Our review highlighted that parent‐based sexual health education intervention is associated with increased parent–adolescent sexual health communication, parental attitudes, and self‐efficacy toward sexual health communication. However, research on such interventions in LMICs, where the rates of HIV and unwanted pregnancies are high, is limited. Moreover, the involvement of fathers in these interventions remains low, reinforcing the stereotype that sexual health education is primarily a woman's responsibility. Although parent‐based sexual education has proven effective [20], there has been a shift toward school‐based CSE [21], often overlooking parental roles. Although the WHO advocates for parental partnership in CSE [15], actual involvement is minimal. There is a need for promoting parental engagement in adolescent sexual health education to improve outcomes and reduce risk behaviors. Our review also revealed variability in intervention types and delivery across different contexts, indicating the necessity for standardized procedures in parent‐based sexual health education interventions. Developing context‐specific curricula for parents, adolescents, educators, and health professionals could enhance effectiveness. We recommend more research in LMICs, including fathers' roles and the long‐term impacts of these interventions on issues such as teenage pregnancy and abortion.

## Author Contributions

The authors confirmed their contribution to the manuscript as follows: **Birhanu Gutu:** study conception, study design, study search, selection and data collection, analysis and interpretation of results, and writing the initial draft. **Abela Mahimbo:** study conception, study design, study search, selection and data collection, analysis and interpretation of results, and manuscript preparation. **Nikki Percival:** study conception, study design, study search, selection and data collection, analysis and interpretation of results and manuscript preparation. **Daniel Demant:** study conception, study design, study search, selection and data collection, analysis and interpretation of results, and critical review of the manuscript. All authors reviewed the results and approved the final version of the manuscript.

## Ethics Statement

The review protocol was registered on the International Prospective Register of Systematic Reviews (PROSPERO) and first published on May 12, 2023, and revised on October 30, 2023, https://www.crd.york.ac.uk/prospero/display_record.php?ID=CRD42023417557.

## Conflicts of Interest

The authors declare no conflicts of interest.

## Supporting information


**Data S1:** psrh70029‐sup‐0001‐Supinfo.

## Data Availability

The authors confirm that the data supporting the findings of this study are submitted with this manuscript as “Supporting Information.”
